# Repressing the repressor: Fra1 controls plasma cell generation

**DOI:** 10.18632/oncotarget.5015

**Published:** 2015-07-20

**Authors:** Dirk Mielenz, Bettina Grötsch, Jean-Pierre David

**Affiliations:** Institute for Osteology and Biomechanics, University Medical Center Hamburg-Eppendorf, Hamburg, Germany

B cell differentiation from the early commitment into the B lymphoid lineage in the bone marrow up to the differentiation into antibody secreting plasma cells is tightly controlled by a transcriptional program dominated by a cascade of repression. Indeed, each step of B cell differentiation to mature B cells appears to depend on transcription factors that, in addition to promoting differentiation, repress key determinants of other hematopoietic lineages or even key regulators of the next or previous steps of B cell differentiation. For instance, Pax5 that is required for early B cell commitment and maintenance of B cell identity acts by repressing the differentiation of lymphoid precursor cells into the other hematopoietic lineages [1]. Globally, key transcriptional regulators of B cell identity such as Pax5, Bcl6 or Bach2, all inhibit the generation of antibody secreting plasma cells by directly or indirectly repressing the transcription of Blimp1 (B lymphocyte-induced maturation protein 1), a transcription factor essential for plasma cell differentiation [2]. Importantly, the progression of activated mature B cells to antibody secreting plasma cells requires to terminate the B cell identity program and to switch on a new transcriptional program driving plasma cells differentiation. Thus, similar to the central role of Pax5 function in early B cell differentiation, the differentiation of B cells into antibody secreting plasma cells as well as their maintenance is tightly controlled by a regulatory network centered on another transcriptional repressor Blimp1 [3]. Indeed, in order to allow plasma cell maturation Blimp1 needs to repress the transcriptional program controlling earlier B cell differentiation (i.e.by switching off Pax5 and Bcl6, for instance) [2]. Of note, these multifaceted potential identities of B cells are accompanied by a very remarkable plasticity of these cells which manifests in a balance of reciprocal but intertwined proliferation-differentiation steps.

Specifically, proliferation of activated B cells that is on the one hand required to induce Blimp1 and to initiate terminal B cell differentation has to be terminated to avoid overshooting immune reactions and to allow terminal differentiation of plasma cells. The molecular control of this particular event has been unclear as it has not been clear whether control of plasma cell differentiation can be uncoupled from the control of B cell proliferation. AP-1 transcription factors formed by heterodimerization of Fos and Jun proteins are known regulators of cell proliferation and apoptosis [4]. The Fos members of AP-1 (c-Fos and Fra1) are known to become quickly up-regulated upon B cell activation [5]. In addition, c-Fos had been shown to promote Blimp1 expression [6]. However, the physiological relevance of these observations was not demonstrated *in vivo*. We recently showed by gain and loss of function experiments that Fra1 enhances activation induced cell death (AICD) upon its induction in activated B cells, and as well limits B cell proliferation [7]. Moreover, transgenic over-expression of Fra1 blocks plasma cell differentiation and immunoglobulin production *in vitro* and *in vivo*. In accordance, mice with B cell-specific deletion of Fra1 show enhanced plasma cell differentiation *in vitro* and *in vivo* as well as exacerbated antibody responses. Interestingly, transgenic Bcl2 overexpression alleviated Fra1 elicited AICD and corrected the B cell proliferation defect. Specifically, *Fra1*tg;*Bcl2*tg B cells could undergo the number of cell divisions required to induce CD138 expression on wild-type B cells. However, Bcl2 could not at all correct the plasma cell differentiation block induced through Fra1, arguing for a specific role of Fra1 in plasma cell differentiation that is independent from its effect on B cell proliferation and survival. Hence it was possible to uncouple proliferation/apoptosis from plasma cell differentiation. Molecularly, Fra1 binds directly to the Blimp1 promoter, represses Blimp1 expression and interferes with binding of the activating AP-1 member c-Fos to the Blimp1 promoter. Accordingly, over-expression of c-Fos in Fra1 transgenic B cells releases Blimp1 repression, arguing for a competitive function of c-Fos and Fra1 in plasma cell differentiation. Therefore, as Fra1 is lacking transcriptional transactivation domains, we propose that Fra1 inhibits Blimp1 expression and plasma cell differentiation through binding to the Blimp1 promoter, thereby, outcompeting the activator c-Fos. Thus, our work suggests that one function of Fra1, by transcriptionally repressing the repressor Blimp1, is to limit plasma cells generation and, by interfering with proliferation, is to prevent overshooting humoral immune responses. Hence, the control of Fra1 abundance is critical for humoral immunity.
